# Ketamine-Treatment During Late Adolescence Impairs Inhibitory Synaptic Transmission in the Prefrontal Cortex and Working Memory in Adult Rats

**DOI:** 10.3389/fncel.2019.00372

**Published:** 2019-08-20

**Authors:** Miguel Ángel Pérez, Camila Morales, Odra Santander, Francisca García, Isabel Gómez, Valentín Peñaloza-Sancho, Pablo Fuentealba, Alexies Dagnino-Subiabre, Pablo R. Moya, Marco Fuenzalida

**Affiliations:** ^1^Laboratorio de Plasticidad Neuronal, Universidad de Valparaíso, Valparaíso, Chile; ^2^Facultad de Ciencias, Centro de Neurobiología y Fisiopatología Integrativa (CENFI), Instituto de Fisiología, Universidad de Valparaíso, Valparaíso, Chile; ^3^Escuela de Ciencias de la Salud, Carrera de Kinesiología, Universidad Viña del Mar, Viña del Mar, Chile; ^4^Centro Interdisciplinario de Neurociencia de Valparaíso, Universidad de Valparaíso, Valparaíso, Chile; ^5^Programa de Doctorado en Ciencias, Mención Neurociencias, Universidad de Valparaíso, Chile; ^6^Laboratorio de Neurogenética, Universidad de Valparaíso, Valparaíso, Chile; ^7^Laboratorio de Neurobiología del Estrés, Universidad de Valparaíso, Valparaíso, Chile; ^8^Laboratory of Neural Circuits, Centro de Neurociencia Universidad Católica, Pontificia Universidad Católica de Chile, Santiago, Chile

**Keywords:** ketamine, late adolescence, PV-INs, GABA, mPFC, schizophrenia-like behavior

## Abstract

Schizophrenia (SZ) is associated with changes in the structure and function of several brain areas. Several findings suggest that these impairments are related to a dysfunction in γ-aminobutyric acid (GABA) neurotransmission in brain areas such as the medial prefrontal cortex (mPFC), the hippocampus (HPC) and the primary auditory cortex (A1); however, it is still unclear how the GABAergic system is disrupted in these brain areas. Here, we examined the effect of ketamine (Ket) administration during late adolescence in rats on inhibition in the mPFC-, ventral HPC (vHPC), and A1. We observe that Ket treatment reduced the expression of the calcium-binding protein parvalbumin (PV) and the GABA-producing enzyme glutamic acid decarboxylase 67 (GAD67) as well as decreased inhibitory synaptic efficacy in the mPFC. In addition, Ket-treated rats performed worse in executive tasks that depend on the integrity and proper functioning of the mPFC. Conversely, we do not find such changes in vHPC or A1. Together, our results provide strong experimental support for the hypothesis that during adolescence, the function of the mPFC is more susceptible than that of HPC or A1 to NMDAR hypofunction, showing apparent structure specificity. Thus, the impairment of inhibitory circuitry in mPFC could be a convergent primary site of SZ-like behavior during the adulthood.

## Introduction

The neocortex undergoes protracted remodeling throughout the transition from adolescence to adulthood (Casey et al., 2008; Hill et al., 2010; Insel, 2010; Gogtay et al., 2011). Diverse environmental factors impinging on the prefrontal cortex (PFC) during adolescence may cause a failure of γ-aminobutyric acid (GABA)-mediated systems to mature (Benes and Berretta, 2001), which could result in susceptibility to several mental illnesses such as schizophrenia (SZ; Marín, 2012). SZ is a disabling brain disorder that starts at early adulthood with presentation of positive, negative, and cognitive symptoms. Positive symptoms include psychotic manifestations, such as hallucinations and delusions. Altered thought and/or behaviors, lack of motivation, severe social withdrawal, and paucity of speech and communication are considered negative symptoms in SZ. Cognitive symptoms include dysfunction in working memory, attention, verbal learning and memory, and executive functions (Lewis et al., 2005; Lewis, 2012). Anatomical and functional studies in *postmortem* tissue from SZ patients have consistently shown changes in GABAergic interneurons (INs) of PFC, hippocampus (HPC) and primary auditory cortex (A1) which may explain some of the symptoms observed in this complex disorder (Stan and Lewis, 2012; Javitt and Sweet, 2015). These changes include lower levels of GABA-synthesizing enzymes GAD67 and GAD65 (Akbarian et al., 1995; Zhang and Reynolds, 2002; Benes et al., 2007; Moyer et al., 2012). Among the different subsets of GABAergic INs, fast-spiking cells that express the calcium-binding protein parvalbumin (PV) seem to be preferentially effected (Benes et al., 1991; Volk et al., 2000; Zhang and Reynolds, 2002; Hashimoto et al., 2003; Fung et al., 2010; Konradi et al., 2011; Wang et al., 2011). The resulting GABA dysfunction in specific brain regions may contribute to some symptoms of SZ (Lisman et al., 2008; Lewis, 2012; Dienel and Lewis, 2018). Indeed, human functional imaging studies suggest that dysfunction of the PFC underlies several deficits associated with SZ, including impaired working memory (WM) (Perlstein et al., 2003). In addition, it has been demonstrated that the reduction of A1 gray matter volume can contribute to auditory processing deficits, which are related to the strengthening of negative and cognitive symptoms of SZ (McCarley et al., 1999; Javitt et al., 2000). Experimental lesions of the PFC in rodents can trigger behavioral alterations related to all three symptomatic categories in SZ (Joel et al., 1997; Murray et al., 2015; Gomes and Grace, 2016). Moreover, it has been shown that the selective inactivation of PV-INs in PFC causes mainly WM impairments (Murray et al., 2015). On the other hand, neuroimaging studies have shown a correlation between increased activity in the human anterior (ventral in rodents) hippocampus (vHPC) and the severity of psychosis (Schobel et al., 2013), while in rats, an increase in the vHPC activity, including disruptions in GABAergic transmission, induces hyperlocomotor activity (Zhang et al., 2002; Taepavarapruk et al., 2008). Therefore, alterations in specific subtypes of GABAergic INs occurring in restricted brain regions may result in distinctive behavioral alterations (Zhang et al., 2002; Murray et al., 2015).

Animal models of SZ have been extensively used in research, especially those that cause a hypofunction of the N-methyl-D-aspartate type glutamate receptor (NMDAR) because its ability to mimic SZ-like symptoms in healthy people (Krystal et al., 1994; Lahti et al., 1995; Malhotra et al., 1996; Newcomer et al., 1999). In rodents, chronic treatment with the NMDAR antagonist ketamine (Ket) during the neonatal period or adulthood reduces immunoreactivity for some GABAergic INs markers such as GAD67 and PV, as well as impairment in WM performance (Becker et al., 2003; Keilhoff et al., 2004; Zhang et al., 2008, 2016; Gastambide et al., 2013; Sabbagh et al., 2013; Jeevakumar et al., 2015; Jeevakumar and Kroener, 2016). However, there is still a lack of understanding of whether there is a dominant brain site for the pathogenesis of the SZ during late adolescence that could impact both GABAergic synaptic transmission and behavior. This is an interesting question, considering that late adolescence is a prodromal period of SZ, when even though subjects do not exhibit typical symptoms, they show cognitive impairment, such as memory and executive dysfunction (Larson et al., 2010; Frommann et al., 2011).

In this study, we administered Ket during late adolescence in rats to verify whether both SZ-like behavior and GABAergic impairments endure during adulthood and if they affect specific brain structures. Here, we report that Ket treatment in rats during late adolescence induced some SZ-like symptoms. Interestingly, we found that only PFC-related behaviors were disrupted, leaving those dependent on vHPC and A1 unaffected. These behavioral changes were correlated with the immunohistochemical and electrophysiological measurements for each region: only mPFC slices from Ket-treated rats exhibited a reduction of GABAergic transmission, accompanied by a lower number of PV+ and GAD67+ cells and reduced immunoreactivity for both proteins. On the other hand, no deficits in A1 or vHPC were found. Therefore, our results support the hypothesis that NMDAR–hypofunction during late adolescence specifically impairs mPFC GABAergic transmission and mPFC–dependent WM during adulthood.

## Materials and Methods

### Animals and Drug Treatments

Male *Sprague–Dawley* rats (vehicle, *n* = 68; ketamine, *n* = 71) were used for all experiments in this study (Supplementary Table S1). Animals were housed in groups of three per cage in a 12/12 light/dark cycle (lights on at 8:00 a.m.). They were maintained in a temperature- and humidity-controlled environment with *ad libitum* access to food and water. On the 45th postnatal day (PND), Ket (30 mg/kg), MK801 (0.1 mg/kg in saline) or vehicle (Veh, NaCl 0.9%) were intraperitoneally (i.p.) applied (total volume 1 mL) for 7 consecutive days according to a modified version of methods described by Becker et al. (2003) and de Oliveira et al. (2011). A single amphetamine (Amph) i.p. dose (1.5 mg/kg, 1 mL) was administered to perform the hyperlocomotion test. All animal procedures were in strict accordance with the Animal care standards outlined in the National Institute of Health (United States) guidelines and were approved by the Institutional Animal Ethics Committee of the Universidad de Valparaíso. Efforts were made to minimize the number of animals used and their suffering.

### Behavioral Tasks

#### Locomotor Activity and Anxiety-Like Behavior

To assess locomotor activity (distance traveled), rats were exposed for 20 min to an open field arena (70 × 70 cm) 30 min after the last Ket injection at 7th day (PND 51) during the adolescent period. In addition, a second assessment of locomotor activity for 5 min was performed during adulthood (PND 60). The total distance traveled and means speeds were determined using video-tracking system ANY-maze (Stoelting Co., Wood Dale, IL, United States). Anxiety was tested in an elevated plus-maze, and the percentage of entries into the open arm was quantified. Both paradigms were used as described in Pérez et al. (2013b).

#### Amphetamine-Induced Behavior

Locomotor activity under Amph effect was assessed in an open field arena illuminated at 30 lux (measured by a digital light intensity meter, Model # MT-4017, Pros’ Kit Instrument Co., Taiwan). To perform this protocol, we used a different cohort of animals (Ket: *n* = 9; Veh *n* = 9). First, the rats were habituated to the open field for 60 min (baseline). After habituation, the animals were injected (i.p.) with 500 μL of saline solution and then placed back into the arena and recorded for the next 60 min. Finally, a single dose of Amph at 1.5 mg/kg was injected, and locomotor activity was recorded for an additional 120 min. The arena was divided into nine regions, on which different parameters were evaluated: the number of transitions (movement from one region to another), distance traveled and time spent in the center and periphery of the arena.

#### Sociability and Preference for Social Novelty Test

Social interaction was measured with the Crawley’s sociability and preference for social novelty test (Kaidanovich-Beilin et al., 2011). Briefly, the apparatus consists of a three-chamber cage, with chambers connected by sliding doors. The first step was habituation, in which the animal can freely move throughout the cage for 5 min. The second step is the sociability test, which consists of placing a second rat (stranger I) in a wire cage in one of the side chambers for 10 min. The third step is the social novelty preference, which consists of placing a third rat (stranger II) in the empty side chamber for 10 min. The time spent in each chamber by the subject rat near stranger I and stranger II in the first and second stage, respectively, was recorded.

#### Spontaneous Alternation in the Y-Maze

Spatial working memory was tested in the Y-maze and measured by a spontaneous alternation task (Hughes, 2004). The Y-maze apparatus was constructed with black plexiglass consisting of 3 equally spaced arms [120°, 41 (L) × 38 (H) × 19 (W) cm each], each containing different visual cues. Rats were placed in one of the arm compartments and allowed to freely explore the maze for 5 min. A spontaneous alternation occurred when the rat entered into a different arm of the maze in each of 3 consecutive arm entries. The percentage of spontaneous alternation was calculated as follows: the number of alternation (entries into three different arms consecutively) divided by the total possible alternations (the number of arm entries -2) × 100 (Miedel et al., 2017).

#### Delayed Non-match-to-Sample Task

PFC-dependent WM was assessed in a T-maze using a delayed non-match-to-sample task (DNMS), which was modified from Wang et al. (2014). The T-maze apparatus was constructed with non-transparent plexiglass and consists of a stem arm [30 (L) × 14 (W) × 22 (H) cm] and two branch arms [30 (L) × 14 (W) × 22 (H) cm each]. Each arm was equipped with a sliding door, allowing connection or isolation of all three arms. The task was based on a reward system; thus, rats were food restricted (20% less than normal consumption) for 48 h before the start of the experiment. Habituation was done for 4 days (5 explorations/day) starting at PND55: rats were placed in the stem arm and allowed to freely explore the entire apparatus for 5 min; food pellets were available at the end of both branch arms. In the second step, rats were trained to perform DNMS, which consisted of 2 trials. The first trial was the “information run” (IR), where one branch arm was blocked, forcing the animal to go into the open arm containing a food reward. The second trial was the “test run” (TR). After the IR, the rat was returned to the stem arm, allowing it to choose between the 2 unblocked branch arms. When the rat chose the previously unvisited arm (correct), it was rewarded with food, whereas if it chose the previously visited arm (incorrect), no reward was given. Training was carried out for 4 days (10 sessions/day) with a delay of 1 min between assays. No more than 3 assays were performed in the same left/right allocation for the IR and TR; the percentage of correct choices > 60% at the 4th day was required to reach the criterion for the following evaluations. On the 9th day, the rats were tested with the WM assay (DNMS), with an interval of 10 s between IR and TR. The percentage of correct choices was measured.

#### Auditory Attention

To measure auditory attention in rats, we used the 2-ACT paradigm (Supplementary Figures S1A,B). Four modular rat operant chambers and accessories (LE1005, LE10022, LE100575, LE100560, Panlab S.L., Barcelona, Spain) were used in the attention task, each within a 67 × 67 × 67 cm^3^ sound-attenuating cubicle lined with 7.5 cm acoustic foam (Vroka S.A., Santiago, Chile). The operant chamber was illuminated to 200 lux (measured by a digital lux meter, Model # LX-1010B, Weafo Instrument Co., Shanghai, China), and the background noise level was 30 dB. During training, the auditory stimuli was delivered through a speaker calibrated with a precision sound level meter (Model # 1100, Quest Technologies, Oconomowoc, WI, United States) to generate 70 dB in the range of 1–15 kHz at the position of the subject. The duration of the auditory stimuli was 0.1 s. The speaker was mounted in front of the 3 nose-pokes, each of which was connected to a liquid dispenser. Operant modules were regulated by the Packwin V1.2 program (Panlab S.L., Barcelona, Spain). All experiments were recorded with an IP camera (VIVOTEK, Sunnyvale, CA, United States) fixed above each operant chamber. Videos were taken using the Nuuo software (Nuuo, Taipei, Taiwan).

Three days after weaning, rats (PND 24) were trained in the behavioral 2-ACT paradigm (Supplementary Figures S1A,B). Animals were water deprived overnight under a protocol approved by the Institutional Animal Ethics Committee of the Faculty of Sciences, Universidad de Valparaíso, Chile. Then, the rat initiated a trial by inserting its nose into the center nose-poke, which triggered the computer to present two types of acoustic stimuli at random, a low-frequency tone of 1 kHz or a high-frequency 15 kHz tone. Rats were trained to respond with right pokes for low tones and left pokes for high tones. Correct trials were rewarded with water. All operant chambers were thoroughly cleaned with a 5% ethanol solution after each trial. Veh and Ket groups were evaluated at the same time. The 2-ACT training had three steps; in each, it was possible to independently analyze learning, memory consolidation and auditory attention. In the first week of training, rats learned to respond with right pokes for low tones and left pokes for high tones (learning period). In the second week, rats were trained in 50 2-ACT trials until reaching 70% correct trials. At the end of that week, rat memory related to 2-ACT was consolidated (memory consolidation period). Beginning in the third week, the rats recalled the task and improved their auditory attention, increasing correct responses to over 80% of trials (auditory attention period). We measured auditory attention three times: (1) One day after the training period (PND 44, Baseline), (2) 1 day after Ket treatment (PND 52), and (3) during adulthood (PND 60) (Supplementary Figure S1A).

#### Immunohistochemistry

For double-labeling of the PV and GAD67 proteins, animals were perfused transcardially with a saline solution (NaCl, 0.9%), followed by 4% paraformaldehyde in 1x phosphate buffer (PBS, pH = 7.4). Brains were postfixed in 4% paraformaldehyde for 24 h at 4°C. Then, the fixed brains were transferred to a 30% sucrose solution (in PBS) containing 0.01% NaN3. Coronal slices (30 μm) were cut on a freezing microtome, collected in 1x PBS and then stored at 4°C. To improve the protein detection the antigen retrieval was performed in citrate acid buffer (10 mM, pH 6.0) at 90°C for 20 min and 20 min followed by 20 min of cooling. Next, the free-floating sections were washed 3 times in 1× PBS, and then the sections were blocked with a solution of 0.3% Triton-X, 1% bovine serum albumin and 1% horse serum in 1× PBS (blocking solution) for 1 h. We used rabbit anti-parvalbumin (Abcam ab11427; 1:750) and mouse anti-GAD67 (Santa Cruz biotechnology, Inc., cat # sc-58531; 1:100) as primary antibodies. Sections were washed at least 3 times for 10 min each in PBS before incubation with the secondary antibodies for 1 h at room temperature. We used Alexa488-conjugated goat anti-rabbit (Jackson immunoresearch cat # 111-545-003; 1:1000) and Cy3-conjugated goat anti-mouse IgG H&L (Jackson immunoresearch cat # 115-165-005; 1:800) in blocking solution. Finally, sections were washed 3 times for 15 min each in PBS and were mounted and cover-slipped using the Fluoroshield mounting medium with 4’,6-diamidino-2-phenylindole (DAPI, ab104139). A minimum of four sections of the mPFC, HPC and A1 for each animal were imaged on a confocal microscope (Nikon eclipse C1) at 20X or 60X. The acquired images were converted to TIFF format, and cell counts for PV, GAD67 and DAPI were performed from the 20x zoom images using EZ-C1 software with the manual function. To measure the average fluorescence intensity of PV and GAD67, we used images obtained at 60X magnification and drew outlines around the soma with the threshold option in ImageJ software (NIH). Finally, the average fluorescence intensity was measured by using the histogram function.

#### Electrophysiological Recordings

Rats were decapitated under deep anesthesia with isoflurane. The brain was quickly removed and submerged in a cold (∼4°C) cutting solution (in mM: 124.00 sucrose, 2.69 KCl, 1.25 KH2PO4, 10.00 MgSO_4_, 26.00 NaHCO_3_, 10.00 glucose). Coronal brain slices (350 μm) were cut with a Vibratome (WPI Instruments, model NVSLM1, Sarasota, Florida, United States) and maintained for > 1 h at room temperature (20–22°C) in artificial cerebrospinal fluid (ACSF). The ASCF for electrophysiological slices recordings was composed as follows in mM: 124.00 NaCl, 2.69 KCl, 1.25 KH_2_PO_4_, 2.00 MgSO_4_, 26.00 NaHCO_3_, 2.00 CaCl_2_, and 10.00 glucose. The pH of the cutting solution and of the ACSF were adjusted at 7.40 with NaOH or HCl and stabilized by bubbling carbogen (95% O_2_, 5% CO_2_). To perform electrophysiological recordings, the slices were transferred into a 2 mL chamber fixed to an upright microscope stage (NIKON, model Eclipse FN1, Tokyo, Japan) equipped with infrared differential interference contrast (DIC) video microscopy and a 40x water immersion objective. The slices were continuously perfused with carbogen-bubbled ACSF (2 mL/min) and maintained at room temperature (22–24°C). D-(-)2-amino-5-phosphonopentanoic acid (D-AP5; 50 μM, Tocris); 7-nitro-2,3-dioxo-1,4-dihydroquinoxaline-6-carbonitrile (CNQX; 20 μM) and tetrodotoxin (TTX; 0.5 μM), were added to the ACSF as needed. All chemicals were purchased from Sigma-Aldrich Chemistry (St. Louis, MO, United States), Tocris (Bioscience, Pittsburgh, PA, United States) and Cayman Chemical (Sarasota, FL, United States).

Whole-cell recordings were performed from the soma of pyramidal neurons in layer II/III pyramidal neurons from the prelimbic cortex (PrL), in CA1 area of vHPC, and A1 cortex, with patch pipettes (4–8 MΩ) filled with an internal solution containing, in mM: 100 Cs-Gluconate, 10 HEPES, 10 EGTA, 4 Na2-ATP, 10 TEA-Cl, and 1 MgCl2-6H2O, buffered to pH 7.2–7.3 with CsOH. Recordings were performed in voltage-clamp configuration using an EPC-7 patch-clamp amplifier (HEKA, Instruments, MA, United States). The holding potential (Vh) was adjusted for PFC and HPC recordings to 0 mV to record inhibitory postsynaptic currents (IPSCs). Moreover, the inhibitory synaptic transmission was also isolated after blocking NMDA- and AMPA-receptors with D-AP5 and CNQX, respectively. In the voltage-clamp configuration, the series resistance was compensated to ∼70%, and neurons were accepted only when the seal resistance was > 1 GΩ and the series resistance (7–14 MΩ) did not change by > 20% during the experiment. The liquid junction potential was measured (∼6 mV) but not corrected. Voltage-clamp data were low-pass filtered at 3.0 kHz and sampled at rates between 6.0 and 10.0 kHz using an A/D converter (ITC-16, InstruTech, MA, United States) and stored with the Pulse FIT software (Heka Instruments). The Pulse Fit program was used to generate stimulus timing signals and transmembrane current pulses. The recording analysis was made offline with pClamp software (Clamp-fit, Molecular Devices). eIPSCs in vHPC were evoked by stimulation and recording in *Stratum pyramidale* (S.P.), while in the PFC and A1 cortex, stimulation and recording were performed in layer II/III. Stimulation was made with a concentric bipolar electrode [60 mm diameter, tip separation ∼100 mm (FHC Inc., ME, United States)]. Averages of IPSCs were obtained by repeated stimulation at 0.3 Hz. We evaluated two forms of short-term synaptic plasticity: paired pulse depression and use-dependent depression. Paired pulse stimulus was applied at four different intervals (30, 70, 100, and 300 ms) and was calculated as R2/R1, where R1 and R2 correspond to peak amplitudes of the first and second eIPSCs, respectively. Use-dependent synaptic depression was analyzed using 10 Hz bursts of 20 stimuli every 60 s (∼3 V, 200 μs). In input-output curves, the stimulus intensity was systematically increased and was different depending on which brain structure we were measuring. Six to ten responses at each intensity were averaged to compute the IPSC amplitude. sIPSC recordings were continuously recorded at 0 mV for 30 min, under CNQX and APV. Additionally, miniature IPSC (mIPSC) was recorded after adding the voltage-gated sodium channel blocker, TTX (0.5 μM), to the bath.

#### Analysis and Statistics

Behavioral data was analyzed with the video-tracking system ANY-maze (Stoelting Co., Wood Dale, IL, United States). Electrophysiological data was analyzed using pClamp software (Molecular Devices Corporation, Chicago, United States). All data are expressed as the mean ± SEM and were analyzed by Student’s *t*-test or two-way repeated measures ANOVA followed by a Bonferroni *post hoc* comparison test, unless otherwise stated. Statistical evaluations were done using Origin 7.0 (Originlab Corporation, Northampton, MA, United States) software. For electrophysiological assays, *n* = number of cells, while for behavioral measures, *n* = number of rats. *P* < 0.05 was accepted as statistically significant; NS = not significant.

## Results

### Schizophrenia–Like Behaviors in Adulthood Are Induced by Ketamine Treatment During Late Adolescence

**FIGURE 1:**
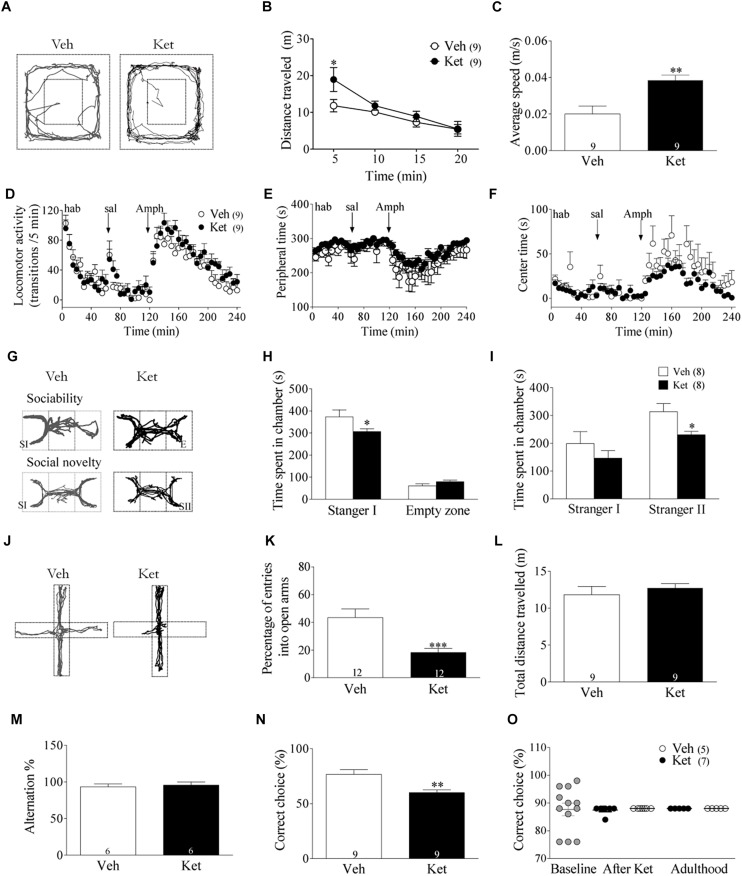
Late Adolescence ketamine treatment induces schizophrenia-like behaviors in adult rats. **(A)** Representative tracking plots from the Veh and Ket group in the open field during the first 5 min (PND 51). **(B)** Ket-treated animals increased the total distance traveled during the first 5 min of exposure to the open field (Repeated measures ANOVA/Bonferroni *post hoc* test, ^∗^*p* < 0.01). **(C)** Hyperlocomotion was accompanied by an increased average speed in the Ket group, *t*-test, ^∗∗^*p* = 0.0039. **(D)** Ket treatment did not change activity transitions/5 min in acute amphetamine application (repeated measures ANOVA/Bonferroni *post hoc* test, *p* > 0.05). **(E,F)** Ket-treated rats did not show different performance in both peripheral time after amphetamine injection or in time of exploration in the anxiogenic central area (repeated measures ANOVA/Bonferroni *post hoc* test, *p* > 0.05). **(G)** Representative tracking plots of Veh-(left) and Ket-treated (right) animals during the assessment of negative symptoms in the three chambers task (SI, stranger 1; E, Empty zone; SII, stranger 2. **(H)** Ket rats displayed impaired explorative behavior during the sociability test, which is observed by a reduction time spent in the chamber containing stranger I compared to the Veh group (two-way ANOVA/Bonferroni *post hoc* test, ^∗^*p* < 0.05). **(I)** Decreased social novelty interest was also observed in Ket rats due the lower time spent in the chamber containing stranger II (two-way ANOVA/Bonferroni *post hoc* test, ^∗^*p* < 0.05). **(J)** Representative tracking plots from Veh and Ket rats after 5 min of exposure to the elevated plus maze (PND 60). **(K)** Ket-treated rats showed a reduction in the percentage of open arm entries compared to the Veh group, *t*-test, ^∗∗∗^*p* < 0.0001. **(L)** Locomotion tested in this paradigm remained unchanged, *t*-test, *p* > 0.05. **(M)** Ket treatment did not change performance in the spontaneous alternation task, showing a similar percentage of spontaneous alternation compared to Veh rats (*t*-test, *p* > 0.05). **(N)** Ket-treated rats showed impaired performance in the delayed-non-match to sample task, observed by a decrease in the percentage of correct choices (*t*-test, ^∗^*p* < 0.01). **(O)** Ket treatment did not change performance for 2-ACT, showing a similar percentage of correct choice during adolescence or adulthood compared to the baseline (paired *t*-test, *p* > 0.05). Data are the mean ± SEM. The number of animals is indicated in parentheses or within the bars. ^∗^*p* < 0.05, ^∗∗^*p* < 0.001, and ^∗∗∗^*p* < 0.0001.

To assess the behavioral consequences of Ket treatment during late adolescence, rats were evaluated in a battery of behavioral tests. Since hyperlocomotion activity has been correlated with positive symptoms of SZ (Lahti et al., 1995), we exposed Veh- and Ket-treated animals to an open field for 20 min after the final injection on the 7th day during the late adolescent period (PND51 in response to Ket). Ket treatment increased the total distance traveled during the first 5 min following exposure to a novel open field (Ket 18.9 ± 3.2 m; Veh 13.2 ± 1.3 m, *p* < 0.05) (Figures 1A,B). The average speed in Ket–treated animals also increased (Ket 0.04 ± 0.01 m/s; Veh 0.02 ± 0.01 m/s, *p* < 0.05) (Figure 1C). In addition, we also evaluated the responsiveness to Amph, since it increases synaptic levels of dopamine and exacerbates psychotic episodes in people with SZ (Bramness et al., 2012), supporting the use of Amph as a model of psychotic symptoms of SZ (Wang et al., 2010). To this end, a group of adult rats (PND60) was used. We observed that during the habituation period and after saline injection, both Veh- and Ket-treated groups showed similar performance based on the number of transitions/5 min (repeated measures ANOVA/Bonferroni *post hoc* test, *P* > 0.05; Figure 1D). Similar results were found after Amph application, where Ket-treated rats displayed no differences in the number of transitions compared to Veh rats (repeated measures ANOVA/Bonferroni *post hoc* test, *P* > 0.05) (Figure 1D) Moreover, to assess anxiety-related behavior in this paradigm, we measured the time spent in the periphery (Figure 1E) and center (Figure 1F) of the open field arena, and we did not find significant differences between groups (repeated measures ANOVA/Bonferroni *post hoc* test, *P* > 0.05). These results indicate that Ket application during adolescence did not alter the responsiveness to Amph in adult rats.

Social behavioral deficits, including social withdrawal and isolation, are frequently observed in SZ patients and are key components of negative symptoms (Couture, 2006). To evaluate these behavioral alterations, the three-chamber social interaction test was used as a proxy in rats (Figure 1G) (Kaidanovich-Beilin et al., 2011). Ket–treated rats demonstrated a significant reduction in time spent in the chamber containing stranger I compared to the Veh group (Ket 306.6 ± 13.9 s; Veh 373.4 ± 31.12 s, *p* < 0.05), which indicates that the sociability of Ket–treated rats is impaired, while time spent exploring the empty zone was similar in both groups (Figure 1H). Subsequently, we examined the preference for social novelty by comparing the choice between the stranger I rat and the stranger II rat (i.e., deficits in social memory) (Moy et al., 2004; van der Kooij and Sandi, 2012; Eagle et al., 2013). Ket– treated rats spent less time exploring in the chambers associated with stranger II compared to the Veh group (Ket 230.5 ± 12.11 s; Veh 314.0 ± 28.8 s, *p* < 0.05) (Figure 1I). In addition, Ket–treated rats traveled significantly less in the chambers containing stranger I and stranger II compared to the Veh group (Supplementary Figures S2E–G). We also found that Ket treatment reduced both the number and duration of contacts to stranger I and stranger II (Supplementary Figures S2I,J) and these rats showed a trend to increase the time spent in self-grooming (Supplementary Figure S2H). Collectively, these results indicate that Ket treatment induces long-lasting social behavior impairments. Next, we tested anxiety-like behaviors in our model. To this end, rats were exposed to the elevated plus-maze during adulthood (Figure 1J), where the Ket–treated group showed a significant decrease in the percentage of open arm entries compared to the Veh group (Ket 43.55 ± 6.01%; Veh 18.26 ± 2.81%, *p* < 0.001) (Figure 1K); no changes were found in total distance traveled (Ket 11.36 ± 1.78 m; Veh 11.82 ± 3.3 m, *p* > 0.05) (Figure 1L). In order to determine the selectivity of our treatment, and considering that Ket, a non-competitive NMDAR antagonist, could also act over other receptors and targets (Sleigh et al., 2014), we evaluated the effect of the selective and non-competitive NMDA receptor antagonist, MK-801 (0.1 mg/kg in saline). In this regard, we found that adult animals treated with MK-801 during late adolescence also exhibited hyperlocomotion and increased anxiety-like behavior (Supplementary Figures S3D–F). In fact, MK-801 treatment increased total distance traveled during the first 5 min following exposure to a novel open field (MK-801 16.8 ± 1.0 m; Veh 11.83 ± 1.6 m, *p* < 0.05) (Supplementary Figures S3A,B). The average speed in MK-801 treated animal was also higher compared to Veh group (MK-801 0.057 ± 0.003 m/s; Veh 0.02 ± 0.004 m/s, *p* < 0.05) (Supplementary Figure S3C). These results reinforce the idea that the SZ-like symptoms observed in our model occurs through an NMDAR-dependent mechanism.

Cognitive dysfunction is considered a clinical feature of SZ (Piskulic et al., 2007). Furthermore, there is considerable evidence suggesting that Ket–treatment during adulthood impairs cognitive function, both in humans and animals (Krystal et al., 1994; Floresco et al., 2009; Smith et al., 2011; Driesen et al., 2013). Thus, to test the effect of Ket–treatment during adolescence over WM in adulthood, we evaluated HPC– and PFC–dependent WM differentially using the Y-maze and T–maze, respectively (Zahrt et al., 1997; Hughes, 2004; Miedel et al., 2017). In the Y–maze spontaneous alternation task, Ket–treated rats alternated between arms at a similar level compared to Veh rats (Ket 89.72 ± 1.9%; Veh 86.9 ± 2.1%, *p* > 0.05) (Figure 1M), suggesting that the HPC-dependent WM is unaltered. However, in the T-maze DNMS task, the Ket–treated rats showed a lower percentage of correct choices (Ket 60.0 ± 2.6%, *n* = 6; Veh 76.7 ± 4.2%, *n* = 6; *p* < 0.05) than the Veh–group (Figure 1N), suggesting that Ket impairs PFC-dependent WM. To verify whether late adolescence Ket treatment could show a differential effect depending on the brain region tested, we additionally evaluated auditory attention, a sensory function that is impaired in SZ (Javitt et al., 2000; Javitt and Sweet, 2015) and whose dysfunction has been related to activity in A1 cortex and could substantially contribute to symptoms and overall impairment in psychosocial outcome (Javitt and Sweet, 2015). To this end, we tested the effect of Ket treatment over auditory attention during both adolescence and adulthood using the 2-ACT task (Pérez et al., 2013a; Supplementary Figures S2A,B). We found that Ket treatment did not affect the percentage of correct choices during both adolescence and adulthood compared to the baseline or Veh group (Figure 1O). Moreover, performance in the 50 trials of the 2-ACT did not reveal any effect of the Ket treatment over DS-correct trials, neither during adolescence nor adulthood, compared to both baseline or Veh group (Supplementary Figures S1A,B), indicating a lack of effect of the Ket treatment over auditory attention or memory consolidation. In addition, the time between trial start and nose poke in correct trials in the 2-ACT did not differ between Ket- and Veh-group compared to the baseline (Supplementary Figures S2C,D). Altogether, these results demonstrate that Ket application in the late adolescence induces SZ-like behaviors in adulthood, mostly because of the impairment of the PFC.

### Late Adolescence Ketamine Treatment During Adolescence Induces Loss of PV and GAD67 Expression in Layer II/III of the mPFC but Not in the CA1 Area of vHPC in Adult Rats

**FIGURE 2:**
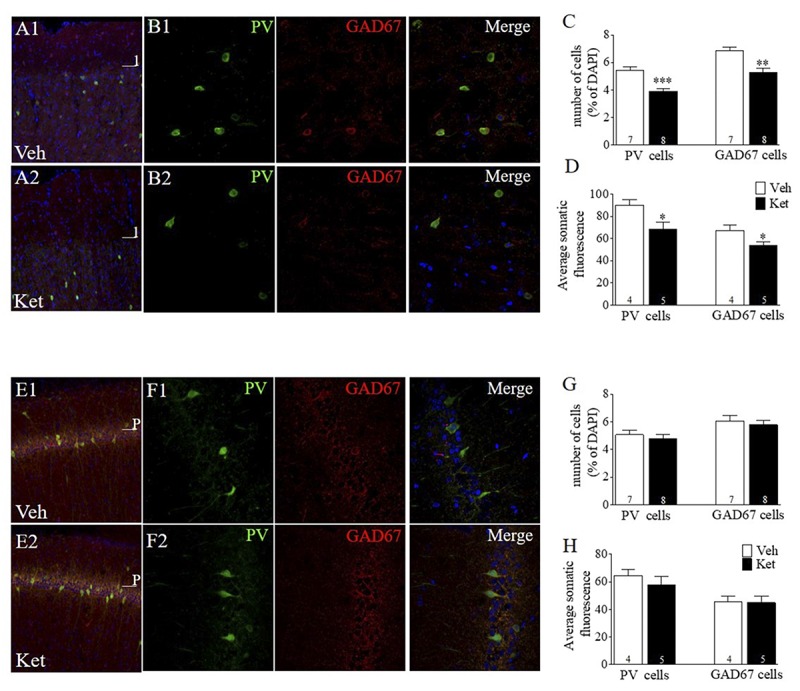
Late Adolescence ketamine treatment reduces the number of PV+ and GAD67+ cells and its immunoreactivity in layer II/III of the mPFC. **(A)** Confocal images of slices from the mPFC stained for parvalbumin (green) and GAD67 (red) from Veh **(A1)** and Ket **(A2)** animals. **(B)** Examples of colocalization of PV, GAD67, and DAPI (blue) in samples from Veh **(B1)** and Ket **(B2)** animals. **(C)** Summary data show that late adolescence Ket treatment significantly reduces the number of both PV cells and GAD67 cells among all DAPI cells in layer II/III of the mPFC compared to the Veh group (*t*-test, ^∗∗^*p* < 0.01). **(D)** Prefrontal brain slices from Ket animals showed a reduction of fluorescence intensity in both PV and GAD67 compared to the Veh group (*t*-test, ^∗^*p* < 0.05). **(E)** Confocal images of slices from CA1 area of vHPC stained for PV (green) and GAD67 (red) from Veh **(A1)** and Ket **(A2)** animals. **(F)** Examples of colocalization of PV, GAD67, and DAPI (blue) in samples from Veh **(F1)** and Ket **(F2)** animals. **(G)** Summary data show that Ket administration did not affect the number of both PV cells and GAD67 cells in the CA1 area of vHPC compared to Veh group (*t*-test, ^∗^*p* > 0.05). **(H)** No changes were detected in both PV and GAD67 fluorescence intensity in Ket-treated animals compared to the Veh group (*t*-test, ^∗^*p* > 0.05). Data are the mean ± SEM. The number of animals is indicated in parentheses or within bars. ^∗^*p* > 0.05, ^∗∗^*p* > 0.001, and ^∗∗∗^*p* > 0.0001.

One of the most consistent findings in *postmortem* brain studies of SZ patients is the decreased expression of INs-related proteins, such as GAD67 (Guidotti et al., 2000; Volk et al., 2000; Hashimoto et al., 2003) and PV (Zhang and Reynolds, 2002; Lewis et al., 2005; Torrey et al., 2005). In addition, Ket treatment during both the neonatal period and adulthood have shown changes in these INs markers (Becker et al., 2003; Keilhoff et al., 2004; Behrens et al., 2007; Jeevakumar and Kroener, 2016; Zhang et al., 2016). However, it is unclear whether Ket treatment during late adolescence alters these INs markers in adulthood. To this end, we performed immunofluorescence assays to evaluate changes in the number of GAD67- and PV+ cells; intensity of the immunoreactive staining was also measured for each protein. Measurements were made in layer II/III of the mPFC, specifically in the PrL area and in the CA1 region of the vHPC (Figure 2). The number of PV+ cells in Ket–treated rats was significantly lower than in the Veh group (Ket 4.12 ± 0.18; Veh 5.74 ± 0.41, *n* = 7; *n* = 8; *p* < 0.001) (Figures 2B,C). We found similar results for the number of GAD67+ cells in Ket–treated animals (Ket 5.11 ± 0.19, Veh 6.52 ± 0.37, *n* = 7; *n* = 8; *p* < 0.05) (Figures 2B,C). Furthermore, we quantified the fluorescence intensity of PV and GAD67 in the somata of INs and found that the median intensity for both PV and GAD67 was lower in Ket-treated animals (PV: Ket 68.26 ± 6.43; Veh 90.01 ± 5.04; GAD67: Ket 53.92 ± 3.27; Veh 67.15 ± 4.68; Figure 2D). Next, we investigated whether the loss of the PV+ and GAD67+ cells observed in the mPFC could also be detected in vHPC, but the two-way ANOVA analysis did not reveal an effect of the treatment in the number of PV cells nor the number of GAD67 cells in the CA1 area in vHPC (Figures 2F,G). Moreover, Ket–treatment did not affect the fluorescence intensity in the somata of PV and GAD67 INs in CA1 region (Figure 2H). These results show that, despite its systemic administration, Ket treatment during adolescence has a differential effect over mPFC and vHPC at the molecular level in adulthood.

### Ket–Treatment Induces Pre- and Postsynaptic Changes in the GABAergic Transmission in the mPFC of Adult Rats

**FIGURE 3:**
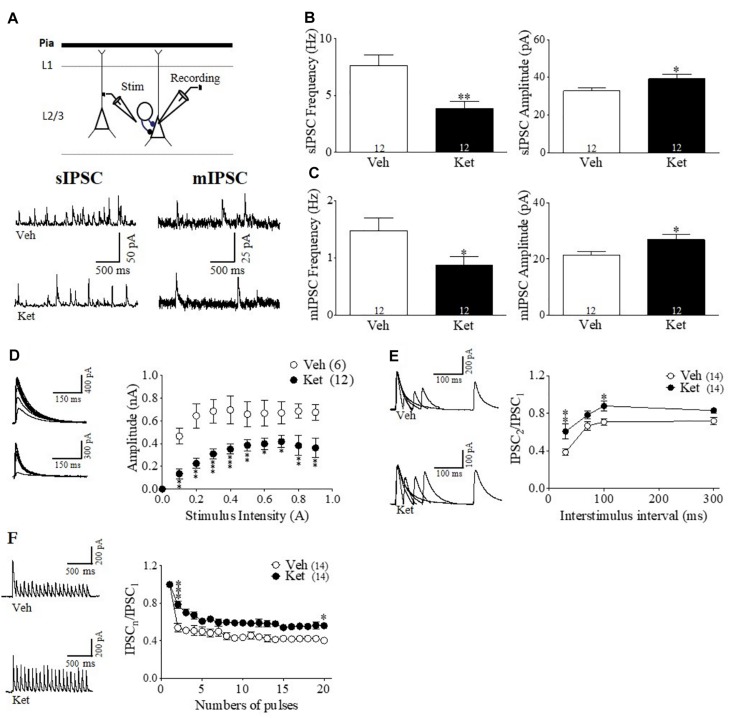
Late Adolescence ketamine treatment induces pre- and postsynaptic changes in GABAergic transmission of the mPFC. **(A)** Schematic arrangement of electrophysiological recordings in layer II/III of the mPFC (upper). Samples traces of sIPSC (left) and mIPSC (right) from Veh and Ket slices (lower). **(B)** Quantitative analyses show that slices from Ket-treated animals have a significant decrease frequency of sIPSC compared to those from Veh rats (left) and increased amplitude of sIPSC (right) (*t*-test, ^∗^*p* < 0.05, ^∗∗^*p* < 0.01). **(C)** Frequency of mIPSC from Ket treatment was also reduced compared to the Veh group (left). Moreover, mIPSC amplitude showed the same direction of change as the sIPSC amplitude (right) (*t*-test, ^∗^*p* < 0.05). **(D)** Sample traces of evoked IPSC amplitudes as a function of stimulus intensity plotted as input/output curves in inhibitory synapses (left). Compared to the Veh group, Ket treatment reduced the amplitude of eIPSC in approximately 20% of IPSC amplitudes at all stimulus intensities (right) (Repeated measures ANOVA/Bonferroni *post hoc* test, ^∗∗∗^*p* < 0.001), sample values of statistical significance: stimulus intensity 0.2A, *p* < 0.01; 0.5A, *p* < 0.05; 0.9A, *p* < 0.05. **(E)** Paired-pulse responses superimposed after subtraction of the first pulse at 30, 70, 100, and 300 ms ISIs (left). Slices from Ket-treated rats showed an increase of paired pulse ratio at intervals equal or lower than 100 ms compared to the Veh group (Repeated measures ANOVA/Bonferroni *post hoc* test, ^∗∗∗^*p* < 0.001). **(F)** Sample traces of synaptic responses evoked by a burst of 20 stimuli at 10 Hz (left). Depression induced by repetitive stimulation showed a reduction in its magnitude for slices from Ket rats compared to the Veh-treated group (right) (Repeated measures ANOVA/Bonferroni *post hoc* test, ^∗∗∗^*p* < 0.001). Data are the mean ± SEM. Number of animals is indicated in parentheses or within bars. ^∗^*p* > 0.05, ^∗∗^*p* > 0.001, and ^∗∗∗^*p* > 0.0001.

In order to test a functional synaptic correlate of the specific impairment of the PFC–dependent WM, we performed whole-cell voltage-clamp recordings from pyramidal neurons in layer II/III of the mPFC obtained from brain slices of Ket– and Veh– treated animals (Figure 3A). Electrophysiological recordings were made here because the majority of the studies have indicated impairments of IN markers in this layer of PFC of SZ patients (Beasley and Reynolds, 1997; Hashimoto et al., 2003; Fung et al., 2010; Lewis, 2012). Initially, we recorded sIPSCs in the presence of CNQX and DAP-5 to isolate the GABAergic transmission. We found a significant reduction in the sIPSC frequency in Ket–treated animals (Ket 3.42 ± 0.46 Hz; Veh 5.34 ± 0.15 Hz, *p* < 0.05; *n* = 12 per condition) (Figures 3A,B left). In addition, we observed a significant increase in the amplitude of the measured sIPSC (Ket 44.58 ± 1.846; Veh 27.84 ± 2.39 pA; *p* < 0.0001; *n* = 12 per condition) (Figure 3B right). MK801 treatment also reduced spontaneous inhibitory events in mPFC, without any effect in vHPC (Supplementary Figures S3G,H). This reduction in sIPSC frequency may be due to a reduction in GABA transmitter release (Castillo, 2012). To estimate efficacy of transmitter release, we performed input-output curves, evaluating eIPSC amplitudes at different stimulus intensities. In this context, the eIPSCs of Ket-treated rats were 20% smaller in amplitude than the ones in Veh-treated rats for the same stimulus intensity (Ket 0.29 ± 0.04 nA; *n* = 12; Veh 0.58 ± 0.06 nA, *n* = 6; *p* < 0.001) (Figure 3D). To investigate whether alterations in inhibitory synaptic transmission were also due to changes in the release probability of INs, miniature IPSC (mIPSC) were recorded after adding the voltage-gated sodium channel blocker, TTX (0.5uM), to the bath. We found a reduction in mIPSC frequency (Ket 0.73 ± 0.12 Hz; Veh 0.88 ± 0.08 Hz, *p* > 0.05; *n* = 12 per condition) (Figure 3C left), which was associated with a significant increase in the amplitude of mIPSC in Ket–treated rats (Ket 32.8 ± 0.91 pA; Veh 21.4 ± 2.14 pA; *p* < 0.001; *n* = 12 per condition) (Figure 3C right). These changes were also replicated by the MK-801 treatment (Supplementary Figures S3J,K). To better understand how the presynaptic component was affected, we evaluated short–term synaptic plasticity through paired pulse depression (PPD) and use-dependent depression protocols (Kaeser et al., 2009). Using the paired pulse protocol, we found that at an interstimulus interval equal to or less than 100 ms, Ket–treatment increased the eIPSC_2_/eIPSC_1_ ratio compared to Veh (30 ms: Ket 0.6063 ± 0.1115; Veh 0.3855 ± 0.04392; *p* < 0.05; 70 ms: Ket 0.7826 ± 0.0434; Veh 0.6666 ± 0.04347; *p* = 0.07; 100 ms: Ket 0.8789 ± 0.9900; Veh 0.7079 ± 0.036, *p* < 0.05; *n* = 14 per condition) (Figure 3E). However, there was no significant change in the eIPSC_2_/eIPSC_1_ at 300 ms interval (*p* > 0.05; *n* = 14 per condition). Also MK801 treatment increase PPR at interval of 30 ms (Supplementary Figure S3I). These results, in addition to the diminished mIPSC frequency, suggest a reduction in the GABA probability of release in Ket–treated animals. Then, to evaluate the ability of GABAergic synapses to recover after repetitive stimulation, we applied a 10 Hz stimulation train, where the eIPSCs amplitude decreased as a result of activity–dependence, short–term depression in both the Veh and Ket group (Figure 3F). During the 10 Hz train stimulation in the Veh group, the amplitude of the 20th response (eIPSC_20_) was reduced by 62% of the first response amplitude (from 1 to 0.38 ± 0.038) (Figure 3F). Although the MK801 did no change the short term depression (Supplementary Figure S3L), Ket treatment displayed less depression of the eIPSC_20_ response, which was reduced by 50% (from 1 to 0.50 ± 0.044), suggesting that in the Ket treatment group, GABAergic synaptic vesicles depleted slower as a consequence of a decrease in the probability of release. These results suggest that compromised GABAergic synaptic transmission could be related to the deficiency in mPFC-dependent WM.

**FIGURE 4:**
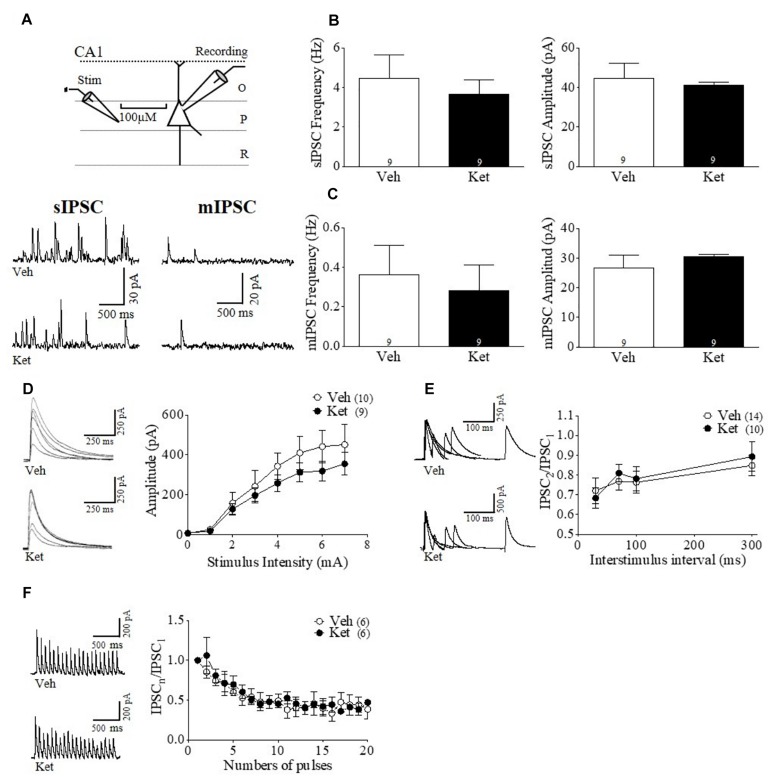
Hippocampal GABAergic transmission during adulthood remains unchanged after chronic late Adolescence ketamine treatment. **(A)** Schematic arrangement of electrophysiological recordings in the pyramidal layer of CA1 area of vHPC (upper). Samples traces of sIPSC (left) and mIPSC (right) from Veh and Ket slices (lower). **(B)** Slices recordings from Ket rats did not show a change of the sIPSC frequency compared to Veh treatment (left). On the other hand, the sIPSC amplitude values were not different between groups (right) (*t*-test, *p* > 0.05). **(C)** Frequency of mIPSC from the Ket group was not significant different compared to the Veh group (left). The mIPSC amplitude was also unchanged after treatment (right) (*t*-test, *p* > 0.05). **(D)** Sample traces of evoked IPSC amplitudes as a function of stimulus intensity plotted as input/output curves in inhibitory synapses (left). Input/output curves revealed that eIPSC amplitudes at all stimulus intensities did not differ between groups (Repeated measures ANOVA/Bonferroni *post hoc* test, *p* > 0.05). **(E)** Paired-pulse responses superimposed after subtraction of the first pulse at 30, 70, 100, and 300 ms ISIs (left). Ket-treated rats did not change paired pulse ratio compared to the Veh group (Repeated measures ANOVA/Bonferroni *post hoc* test, *p* > 0.05). **(F)** Sample traces of synaptic responses evoked by a burst of 20 stimuli at 10 Hz (left). Depression induced by repetitive stimulation remained unchanged in slices from Ket rats compared to the Veh-treated group (right) (Repeated measures ANOVA/Bonferroni *post hoc* test, *p* > 0.05). Data are the mean ± SEM. Number of animals is indicated in parentheses or within bars. ^∗^*p* > 0.05, ^∗∗^*p* > 0.001, and ^∗∗∗^*p* > 0.0001.

Although our immunohistochemistry and behavioral results do not suggest an effect by late adolescent Ket treatment in the vHPC, it is formally possible that GABAergic transmission may still be disrupted in this brain region. Thus, to determine whether Ket treatment modifies hippocampal GABAergic synaptic transmission, we performed whole–cell voltage–clamp recordings from pyramidal neurons found in CA1 area of the vHPC (Figure 4). We did not find any differences between the groups in either the frequency or amplitude of sIPSCs (Ket 4.06 ± 0.78 Hz; Veh 4.22 ± 1.04 Hz; Ket 28.89 ± 2.46 pA Veh 33.16 ± 5.90 pA; *n* = 9 per conditions) (Figure 4B). The frequency and amplitude of mIPSCs were also unchanged (Ket 0.27 ± 0.038 Hz; Veh 0.42 ± 0.092 Hz; Ket 19.81 ± 0.92 pA; Veh 18.21 ± 1.52 pA; *n* = 9 per conditions) (Figure 4C). Accordingly, we found that MK801 treatment also did not affect the frequency or amplitude of both sIPSCs and mIPSCs (data not shown). Moreover, input/output experiments revealed that Ket treatment did not change the eIPSC amplitudes at all stimulus intensities (Figure 4D). Finally, neither the paired pulse protocol at different intervals (Figure 4E) nor the GABAergic depression induced by repeated stimulation were different in Ket–treated animals compared to the Veh group (Figure 4F).

**FIGURE 5:**
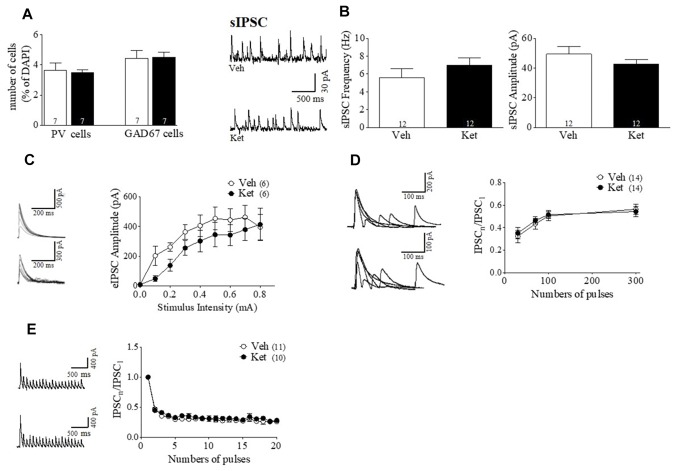
Inhibitory markers and GABAergic transmission during adulthood remains unchanged in auditory cortex after ketamine treatment during adolescence. **(A)** Summary data show that Ket administration did not affect the number of both PV cells and GAD67 cells in layer II/III of A1 compared to Veh group (*t*-test, *p* > 0.05). **(B)** Samples traces of sIPSC (left) from Veh (upper)- and Ket-slices (lower). Whole cell recordings from Ket rats did not show a change of the sIPSC frequency or amplitude compared to Veh-treatment (*t*-test, *p* > 0.05) (right). **(C)** Sample traces of evoked IPSC amplitudes as a function of stimulus intensity plotted as input/output curves at inhibitory synapses (left). Input/output curves revealed that eIPSC amplitudes at all stimulus intensities do not change by Ket treatment (right) (Repeated measures ANOVA/Bonferroni *post hoc* test, *p* > 0.05). **(D)** Paired-pulse responses superimposed after subtraction of the first pulse at 30, 70, 100, and 300 ms ISIs (left). Ket-treated rats did not change paired pulse ratio compared to Veh group (Repeated measures ANOVA/Bonferroni *post hoc* test, *p* > 0.05). **(E)** Sample traces of synaptic responses evoked by a burst of 20 stimuli at 10 Hz (left). Depression induced by repetitive stimulation remained unchanged in slices from Ket rats compared to Veh-treated group (right) (Repeated measures ANOVA/Bonferroni *post hoc* test, *p* > 0.05). Data are mean ± SEM. Number of animals is indicated in parentheses or within bars. ^∗^*p* > 0.05, ^∗∗^*p* > 0.001, and ^∗∗∗^*p* > 0.0001.

Meta-analysis studies have demonstrated a reduction in cortical gray matter volume, particularly in the prefrontal and temporal cortices in patients with SZ (Shenton et al., 2001). However, even when we did not find an effect of the Ket treatment over the auditory function-depending behavioral task, we sought to determine whether Ket had an effect at the molecular and cellular level in another cortical area. As in the mPFC, we measured the interneurons markers (PV and GAD67) and characterized the inhibitory synaptic transmission onto pyramidal neurons in layer II/III of A1. Specifically, A1 cortex was chosen because this area is involved in auditory sensory function by discriminating tones frequencies we hear, a sensory function that is altered in patients with SZ (Javitt et al., 2000; Leitman et al., 2010), and it is thought to contribute to negative and cognitive symptoms (Moyer et al., 2012). Immunofluorescence assays showed no differences in the number of cells positive for PV (Ket 3.49 ± 0.17, *n* = 7; Veh 3.64 ± 0.48, *n* = 7; *p* > 0.05) (Figure 5A) nor for GAD67 in this brain area (Ket 4.49 ± 0.33, *n* = 7; Veh 4.43 ± 0.52, *n* = 7; *p* > 0.05) (Figure 5A). Then, we characterized inhibitory synaptic transmission as we did in the mPFC and vHPC. In this case, the sIPSC of A1 slices remained unchanged after Ket treatment, both in frequency (Ket 6.96 ± 0.83 Hz; Veh 5.59 ± 0.98 Hz) and amplitude (Ket 42.68 ± 2.99 pA; Veh 49.44 ± 5.08 pA; *n* = 12 per conditions) (Figure 5B). Similarly, the input-output curves (Figure 5C), PPD and use-dependent depression (Figures 5D,E) were not different between groups.

Collectively, our results highlight the fact that, despite the systemic application of Ket, its effect is restricted to the mPFC, whose maturation is later in the developmental timeline after the adolescence period in mammals (Insel, 2010).

## Discussion

This study provides the first evidence that late adolescence Ket treatment is able to induce mPFC impairment in a specific manner and that PFC dysfunction alone is sufficient to induce the SZ-like behavior. We found that chronic exposure to the NMDAR antagonist Ket or MK-801, during the late adolescent period, resulted in long-lasting changes in behavior during adulthood, such as anxiety-like behavior, social withdrawal, and impairment of the PFC-dependent WM. In addition, at the molecular and cellular level, reduced expression of PV+ and GAD67+ was accompanied by GABAergic impairment in the mPFC. Notably, the adult CA1 area of vHPC and layer II/III of A1 were not affected by the same Ket treatment. Together, our results provide a strong experimental support for the hypothesis that PFC functions are more susceptible to disruption by NMDAR hypofunction. Thus, mPFC could be a key susceptibility region in the pathogenesis of SZ during the late adolescent period of neurodevelopment, in which the PFC undergoes greatest changes in its morphology and synaptic connectivity. Importantly, compared to the vHPC, the maturation of cortical areas occur later in development (Casey et al., 2008). Indeed, the cortical circuitry of adolescent rats is still in development during the time our Ket treatment and, therefore, it is susceptible to external stimuli (Jaaro-Peled et al., 2009; Insel, 2010; Hoftman and Lewis, 2011; Caballero and Tseng, 2016). Alternatively, differences on susceptibility to oxidative damage could be another explanation for the apparent specific brain impairment. Ket administration can potentiate oxidative stress (Reus et al., 2017), and it has been demonstrated that the PFC is more susceptible to oxidative damage than the HPC (Fowler et al., 2014; Zlatković et al., 2014). Moreover, oxidative stress is negatively correlated with the integrity of PV-INs (Steullet et al., 2017). Thus, higher oxidative stress in the PFC could be related to PV+ impairment and the behavioral and cognitive dysfunction associated with SZ. A loss of PV+ expression is a hallmark of the NMDAR hypofunction models (Behrens et al., 2007; Zhang et al., 2008) because it mimics changes in the subset of PV-INs observed in the postmortem brains of patients with SZ (Lewis et al., 2005; Akbarian and Huang, 2006). We found that Ket-treated animals during late adolescence showed a decreased number and intensity of PV and GAD67 immunoreactivity in layer II/III of the mPFC in adulthood. Fast-spiking firing pattern PV-INs exert a powerful control over the excitability of principal neurons (Markram et al., 2004; Fuchs et al., 2007). They are also essential for the generation of brain gamma oscillations (Bartos et al., 2002; Cardin et al., 2009), which appear to be a critical mechanism underlying high-order cognitive functions of mPFC and HPC (Howard et al., 2003; Cho et al., 2006; Yamamoto et al., 2014). Thus, a reduction in PV-INs number and immunoreactivity in the mPFC may underlie the cognitive disturbances associated with SZ (Beasley and Reynolds, 1997). Indeed, we found that Ket treatment induced deficits in PFC-dependent WM. Conversely, we did not find similar changes in the vHPC or A1. These results suggest a strong relationship between disruption in WM and GABAergic changes, which is seen in SZ patients (Starc et al., 2017).

One of the most highly replicated findings in animal models of SZ using NMDAR antagonists, such as phencyclidine, Ket or MK-801, is the ability of these drugs to induce several SZ-like symptoms seen in humans (Behrens et al., 2007; du Bois and Huang, 2007; Zhang et al., 2008). Psychosis, one of the most characteristic manifestations in positive symptoms, can be explained by dopaminergic hyperfunctioning- which is why dopaminergic agonists have also served as animal models for positive symptoms of SZ (Lodge and Grace, 2007; Bubeníková-Valesová et al., 2008). Increases in dopaminergic function can be evaluated by measuring locomotor activity either in humans or rodents (Bubeníková-Valesová et al., 2008; Minassian et al., 2010). Furthermore, it has been speculated that the loss of GABAergic neurons and the resulting disinhibition in the PFC and vHPC may lead to desynchronization and dysfunctional regulation of the dopaminergic system (Lisman et al., 2008; Kolata et al., 2018). We found novelty-induced hyperlocomotion in Ket–treated animals during the first 5 min of spontaneous exploration in an open field-test performed 30 min after the last injection of Ket during the late adolescence. In addition, previous studies demonstrated that the NMDAR blockade with MK-801 at the early postnatal stage did not affect novelty–induced spontaneous locomotor activity during adulthood (Harris et al., 2003; Stefani and Moghaddam, 2005). These results highlight important differences in the effects of NMDAR antagonist administration at different postnatal stages. Also, we measured the behavioral responsiveness to Amph in adulthood, which is used to increase dopaminergic activity and, as a consequence, hyperlocomotion (Uehara et al., 2010; van den Buuse, 2010). We observed that Ket-treated animals did not increase locomotor activity in response to an acute dose of Amph 1.5 mg/kg. Interestingly, Jeevakumar and colleagues (Jeevakumar et al., 2015) found that Amph-induced behavior during adulthood was not different between early postnatal Ket-treated mice and the Veh-treated group at Amph doses of 1.0 and 2.5 mg/kg, while for a 5 mg/kg dose locomotion of Ket-treated rats was lower compared to Veh group. However, Uehara et al. (2010) found that methamphetamine induced hyperlocomotion in adult rats treated with MK-801 during early postnatal stage. These results might suggest that the behavioral response to Ket/Amph vs. MK-801/Amph could depend on the strain, doses of Amph, developmental stage, as well as the level of anxiety when the animal is performing the tasks. Thus, Ket administration during the late adolescent period appears to be a useful model specifically for testing the cognitive and negative symptoms of SZ, which can be exacerbated by impairment of A1 cortex (Javitt et al., 2000; Leitman et al., 2010). It has been demonstrated that an aberrant hippocampal activity may impair dopaminergic activity, which might underlie the increased locomotor response to Amph (Harrison, 2004; Lodge and Grace, 2007). We observed that Ket treatment during the late adolescent period does not affect GABAergic transmission PV expression or GAD67 levels in adult vHPC. These results suggest that the lack of Amph–induced psychomotor behavior observed in our study could be explained by normal hippocampal functioning.

Changes in GABAergic transmission induced by Ket appear to show structural specificity. While we did not observe alterations of the inhibitory synaptic transmission over pyramidal neurons in both the CA1 area of the vHPC or layer II/III of A1 cortex, we found significant changes in the GABAergic synaptic efficacy in pyramidal neurons in layer II/III of mPFC. Certainly, chronic exposure to Ket during late adolescent period resulted in long–term impairments of the GABAergic synaptic transmission, which are directly associated with modification of short- and long-lasting changes in synaptic plasticity at excitatory synapses (Abbott and Nelson, 2000; Chevaleyre and Piskorowski, 2014). Our data showed that adolescence treatment with Ket on GABAergic efficacy appears to be presynaptic, as suggest by the decrease of (1) PPR, (2) the frequency of both sIPSC and mIPSC and use-dependent depression. The reduced sIPSC frequency could be related to a decrease of IN excitability. Also, a reduction in frequency of miniature events could occur by release failures (Chen and Roper, 2003). Indeed, short-term depression has been attributed to an increased neurotransmitter release probability from presynaptic terminals (O’Donovan and Rinzel, 1997). By analyzing two forms of short–term synaptic plasticity, paired pulse depression (PPD) and use-dependent depression during a 10 Hz stimulus train. We found evidence for a decrease in GABA neurotransmitter release. In input-output curves, we found that for similar stimulus intensities the Ket-treated mPFC slices exhibited lower IPSC amplitude than control slices, suggesting that Ket treatment diminished evoked inhibitory synaptic transmission. Surprisingly, we observed that Ket treatment increased the amplitude of both sIPSC and mIPSC, which might be the result of Ket-induced changes of GABA vesicular content, postsynaptic sensitivity, or both. It has been shown that in layer II/III of individuals with SZ, GABA_*A*_ α2 subunit mRNA levels are elevated, which could explain this increase in the amplitude of the sIPSC and mIPSC (Volk et al., 2002). The increase in postsynaptic GABA_*A*_ α2 receptor protein levels has been interpreted as coordinated compensations in response to a PV deficit in synthesis and release of GABA to regain normal GABA signaling (Lewis et al., 2005; Beneyto et al., 2011). In addition, the reduction in PV and GAD67 levels that result from chronic NMDAR blockade are believed to represent maladaptive mechanisms in a faulty effort to maintain a correct excitatory/inhibitory in the cortical network (Behrens et al., 2007; Lisman et al., 2008). Interestingly, it has been shown that NMDA receptors are essential to regulate the peri-adolescent maturation of the GABAergic networks (Thomases et al., 2013). Thus, it is possible that NMDA antagonist Ket/MK-801 treatment may impede the normal function of GABA synaptic efficacy.

Collectively, our data show that NMDAR antagonist Ket and MK801 administration during late adolescence impairs in a specific manner PFC over HPC or A1 cortex, and that this discrete mPFC disruption is enough to induce SZ-like behaviors and corresponding changes on PV and GAD67 levels, as well as GABAergic impairment on layer II/III of the mPFC. Therefore, administration of Ket during the late adolescent period appears to be a useful model for cognitive and negative symptoms of SZ. Moreover, reductions in PV and GAD67 in the mPFC are related to an impairment of the inhibitory synaptic transmission. The mechanisms underlying late adolescent impairment in the PFC and their potential relationship to the pathophysiology of SZ clearly warrants further investigation.

## Data Availability

The datasets generated for this study are available on request to the corresponding author.

## Ethics Statement

The Institutional Animal bioethics Committee for research with experimental animals (CIBICA), Universidad de Valparaío declares that he has evaluated the protocol of experimentation used in the present work entitled: Ketamine-treatment during late adolescence impairs inhibitory synaptic transmission in the prefrontal cortex and working memory in adult rats of the researcher, Dr. Marco Fuenzalida Núñez, University of Valparaíso. The Institutional Animal bioethics Committee of the Universidad de Valparaíso and according to standards outlined in the National Institute of Health (United States) guidelines approves the experimental protocol used in this investigation.

## Author Contributions

MP, CM, OS, FG, IG, and VP-S conducted the experiments and data analysis. AD-S, PF, PM, and MF contributed to experimental design, data discussion, and writing.

## Conflict of Interest Statement

The authors declare that the research was conducted in the absence of any commercial or financial relationships that could be construed as a potential conflict of interest.

## Supplementary Material

FIGURE S1 Schematic drawing of the experimental design, structure of the basic two-alternative choice task (2-ACT), and locomotor activities during adulthood. **(A)** The arrow represents the postnatal days of the animals (PND). The behavioral tasks and electrophysiological tools were measured during adulthood between PND 60 and 70. The locomotor activities in reaction to Ket were done during adolescence (PND 51). **(B)** With auditory attention training beginning after weaning, the rats were trained in the 2-ACT for 20 days. The rat initiated a trial by inserting its nose into the center nose-poke, which triggers the computer to present two types of acoustic stimuli at random: one was a low-frequency tone at 1 kHz, and the other was a high-frequency 15 kHz tone. The rats were trained to respond with right pokes for low tones and left pokes for high tones. To analyze the ketamine effects on auditory attention, the animals were subjected to 50 2-ACT trials 1 day after 2-ACT training (baseline) during adolescence (1 day after Ket treatment, PND 51) and during adulthood (PND 60).

FIGURE S2 Auditory attention and decision-making remains unchanged after ketamine treatment during adolescence and tests for sociability and social novelty preference. A difference score was then determined by subtracting the correct trials after Ket treatment during adolescence or adulthood from those obtained during the baseline (DS-correct trials). **(A)** During adolescence, Ket treatment did not affect the DS-correct trials through the 50 trials of the 2-ACT (left). A summary of DS-correct trials during adolescence shows that Ket treatment did not change the auditory attention (Veh 1.75 ± 3.22; Ket-1.16 ± 2.3, *p* > 0.05) (right). **(B)** During adulthood, Ket treatment did not affect the performance of correct trials of the 2-ACT (left). A summary of the DS-correct trials during adulthood shows that Ket treatment did not change the auditory attention (Veh -0.25 ± 1.79; Ket 0.33 ± 2.41, *p* > 0.05) (right). The time used for decision-making to perform each correct trial in the 2-ACT did not change during the 50 trials during adolescence **(C)** or during adulthood **(D)** after Ket treatment. **(E)** Experimental schemes for sociability and social preferences of adult male rats in the three-chamber apparatus. **(F)** Distance traveled in the chamber containing stranger I compared to the Veh group (two-way ANOVA/Bonferroni *post hoc* test, ^∗^*p* < 0.05). **(G)** Preference for a novel stranger (stranger 2) vs. the first unfamiliar mouse (stranger 1). Decreased social novelty interest was also observed in Ket rats due the lower distance traveled in the chamber containing stranger II (two-way ANOVA/Bonferroni *post hoc* test, ^∗^*p* < 0.05). **(H)** The time spent in self-grooming shows trends to increase in the Ket group compared to Veh group (two-way ANOVA/Bonferroni *post hoc* test, *p* > 0.05). **(I)** Ket shows trends to reduce the number of contacts spent near to the stranger I and II compared to Veh group (two-way ANOVA/Bonferroni *post hoc* test, *p* > 0.05). **(J)** Ket reduce significantly the duration of contacts spent near to stranger I compared to Veh group (two-way ANOVA/Bonferroni *post hoc* test, ^∗^*p* < 0.05). The data are represented by the mean ± SEM.

FIGURE S3 MK801 treatment during late adolescent impair both the behavioral and inhibitory synaptic transmission in the prefrontal cortex. **(A)** Representative tracking plots from the Veh and MK801 group in the open field during the first 5 min during adulthood (PND60). **(B)** MK801 treatment increased the total distance traveled during the first 5 min of exposure to the open field (*t*-test, ^∗^*p* < 0.01). **(C)** Hyperlocomotion was accompanied by an increased average speed in the MK801 group, *t*-test, ^∗∗∗^*p* = 0.0001. **(D)** Representative tracking plots from Veh and Ket rats after 5 min of exposure to the elevated plus maze. **(E)** MK801 treatment induces a reduction in the percentage of open arm entries compared to the Veh group, *t*-test, ^∗^*p* < 0.01. **(F)** The total distance traveled tested in this paradigm remained unchanged, *t*-test, *p* > 0.05. **(G)** Quantitative analyses show that slices from MK801 treatment have a significant decrease frequency of sIPSC compared to those from Veh rats and increased amplitude of sIPSC **(H)** (*t*-test, ^∗^*p* < 0.05). **(I)** Paired-pulse responses superimposed after subtraction of the first pulse at 30, 70, 100, and 300 ms ISIs. Slices from MK801treatment rats showed an increase of paired pulse ratio at intervals equal to 30 ms compared to the Veh group (Repeated measures ANOVA/Bonferroni *post hoc* test, ^∗^*p* < 0.05. **(J)** Frequency of mIPSC from MK801 treatment was also reduced compared to the Veh group. Moreover, mIPSC amplitude showed a significant increase **(K)** (*t*-test, ^∗^*p* < 0.05). **(L)** Synaptic responses evoked by a burst of 20 stimuli at 10 Hz. Depression induced by repetitive stimulation did not changes in its magnitude for slices from MK801 treatment compared to the Veh-treated group (Repeated measures ANOVA/Bonferroni *post hoc* test, *p* > 0.05). Data are the mean ± SEM. Number of animals is indicated in parentheses or within bars.

TABLE S1 Number of animals used in each experiment: the table shows the total animals used in each experiment.

## Abbreviations

ACSF: artificial cerebrospinal fluid
A1: Primary auditory cortex
GABA: γ-aminobutyric acid
Amph: amphetamine
ANOVA: analysis of variance
CNQX: 7-nitro-2,3-dioxo-1,4-dihydroquinoxaline-6-carbonitrile
D-AP 5: 2-amino-5-phosphonopentanoic acid
EPSCs: excitatory postsynaptic currents
GAD67: glutamic acid decarboxylase 67
HPC: hippocampus
IPSCs: inhibitory postsynaptic currents
Ket: ketamine
mPFC: medial prefrontal cortex
mIPSC: miniature inhibitory postsynaptic currents
MK-801: Dizocilpine
NMDAR: N-methyl -D-aspartate receptor
PV+: calcium-binding protein parvalbumin
PFC: prefrontal cortex
PND: postnatal days
PPR: paired-pulse ratio
SZ: schizophrenia
sIPSC: spontaneous inhibitory postsynaptic currents
TTX, tetrodotoxin. Veh: vehicle
WM: working memory.
