# Supplementary figures and images for: Ketamine-Treatment During Late Adolescence Impairs Inhibitory Synaptic Transmission in the Prefrontal Cortex and Working Memory in Adult Rats
Source: Front Cell Neurosci. 2019 Aug 20;13:372. doi: 10.3389/fncel.2019.00372 (PMC6710447; doi:10.3389/fncel.2019.00372)

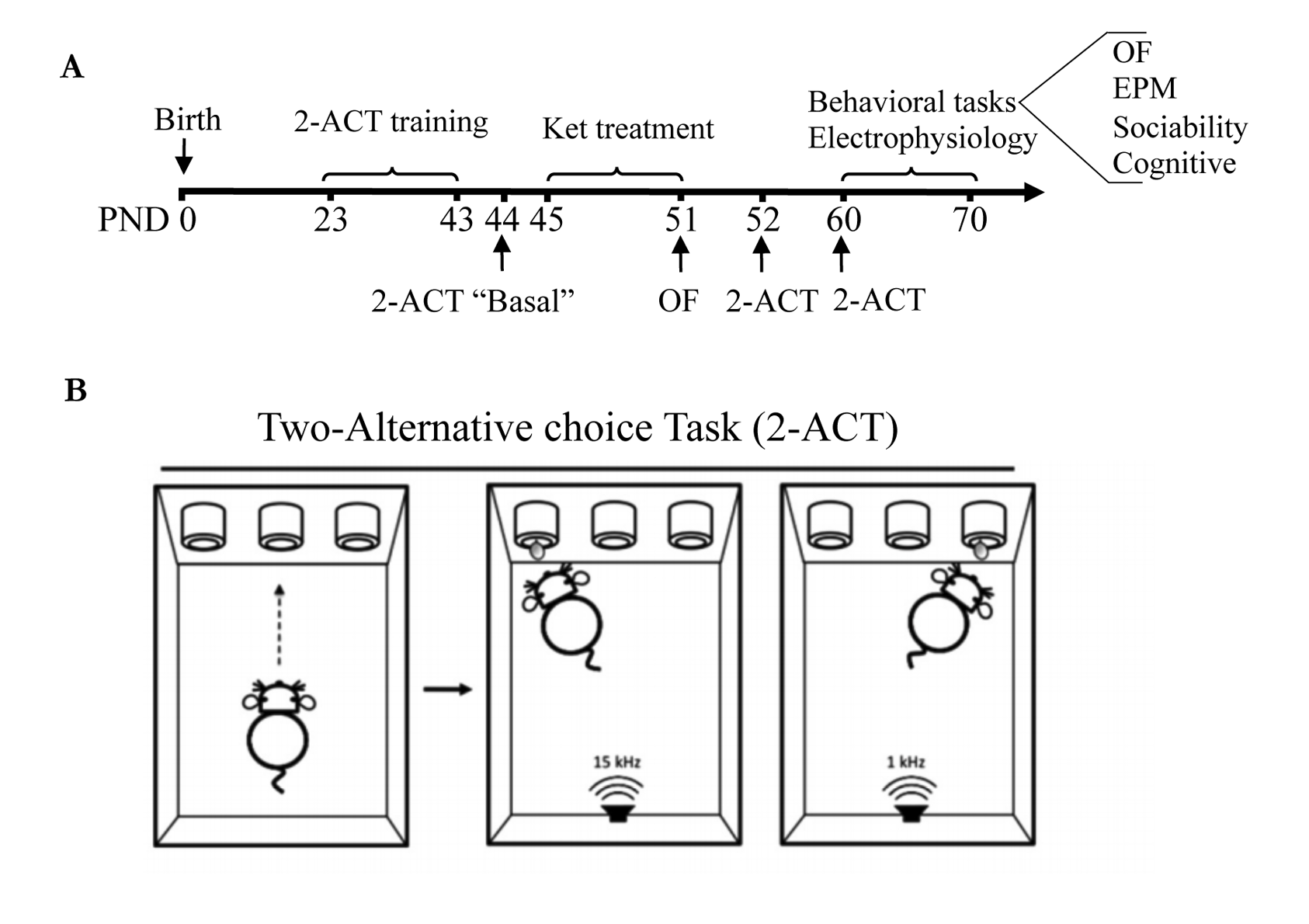

Supplement: FIGURE S1 — Schematic drawing of the experimental design, structure of the basic two-alternative choice task (2-ACT), and locomotor activities during adulthood. (A) The arrow represents the postnatal days of the animals (PND). The behavioral tasks and electrophysiological tools were measured during adulthood between PND 60 and 70. The locomotor activities in reaction to Ket were done during adolescence (PND 51). (B) With auditory attention training beginning after weaning, the rats were trained in the 2-ACT for 20 days. The rat initiated a trial by inserting its nose into the center nose-poke, which triggers the computer to present two types of acoustic stimuli at random: one was a low-frequency tone at 1 kHz, and the other was a high-frequency 15 kHz tone. The rats were trained to respond with right pokes for low tones and left pokes for high tones. To analyze the ketamine effects on auditory attention, the animals were subjected to 50 2-ACT trials 1 day after 2-ACT training (baseline) during adolescence (1 day after Ket treatment, PND 51) and during adulthood (PND 60). [file Image_1.tiff]

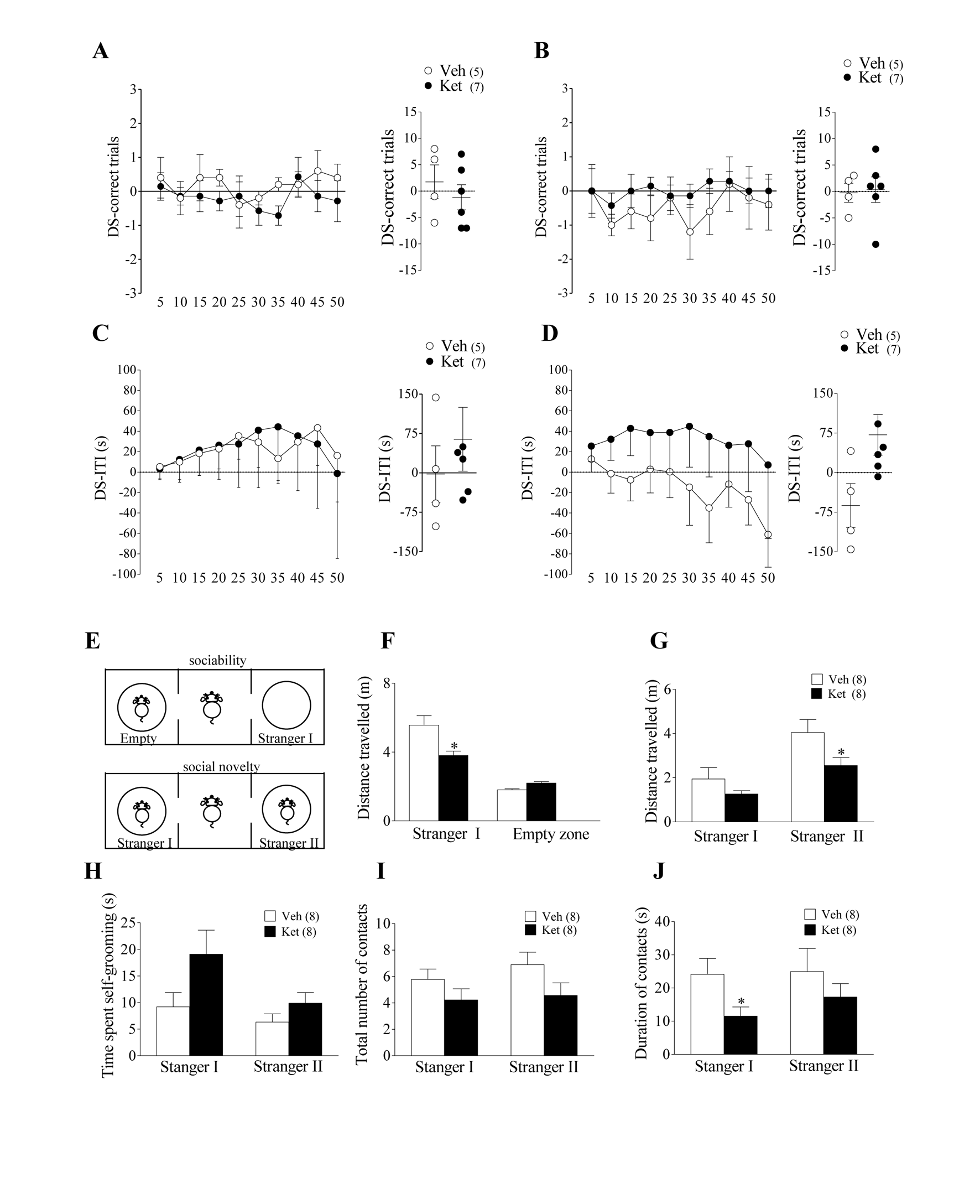

Supplement: FIGURE S2 — Auditory attention and decision-making remains unchanged after ketamine treatment during adolescence and tests for sociability and social novelty preference. A difference score was then determined by subtracting the correct trials after Ket treatment during adolescence or adulthood from those obtained during the baseline (DS-correct trials). (A) During adolescence, Ket treatment did not affect the DS-correct trials through the 50 trials of the 2-ACT (left). A summary of DS-correct trials during adolescence shows that Ket treatment did not change the auditory attention (Veh 1.75 ± 3.22; Ket-1.16 ± 2.3, p > 0.05) (right). (B) During adulthood, Ket treatment did not affect the performance of correct trials of the 2-ACT (left). A summary of the DS-correct trials during adulthood shows that Ket treatment did not change the auditory attention (Veh -0.25 ± 1.79; Ket 0.33 ± 2.41, p > 0.05) (right). The time used for decision-making to perform each correct trial in the 2-ACT did not change during the 50 trials during adolescence (C) or during adulthood (D) after Ket treatment. (E) Experimental schemes for sociability and social preferences of adult male rats in the three-chamber apparatus. (F) Distance traveled in the chamber containing stranger I compared to the Veh group (two-way ANOVA/Bonferroni post hoc test, ∗p < 0.05). (G) Preference for a novel stranger (stranger 2) vs. the first unfamiliar mouse (stranger 1). Decreased social novelty interest was also observed in Ket rats due the lower distance traveled in the chamber containing stranger II (two-way ANOVA/Bonferroni post hoc test, ∗p < 0.05). (H) The time spent in self-grooming shows trends to increase in the Ket group compared to Veh group (two-way ANOVA/Bonferroni post hoc test, p > 0.05). (I) Ket shows trends to reduce the number of contacts spent near to the stranger I and II compared to Veh group (two-way ANOVA/Bonferroni post hoc test, p > 0.05). (J) Ket reduce significantly the duration of contacts spent near [file Image_2.TIF]

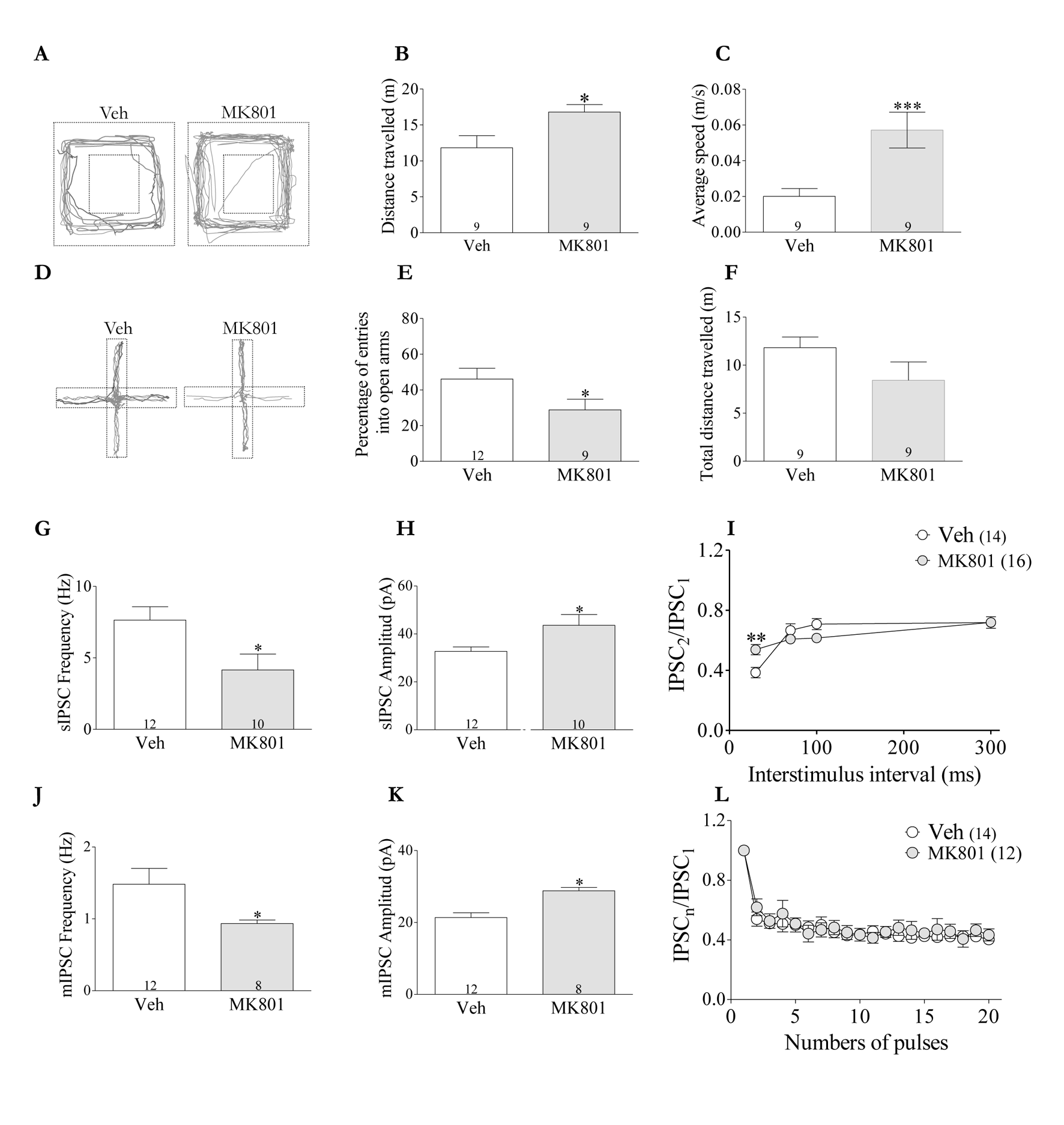

Supplement: FIGURE S3 — MK801 treatment during late adolescent impair both the behavioral and inhibitory synaptic transmission in the prefrontal cortex. (A) Representative tracking plots from the Veh and MK801 group in the open field during the first 5 min during adulthood (PND60). (B) MK801 treatment increased the total distance traveled during the first 5 min of exposure to the open field (t-test, ∗p < 0.01). (C) Hyperlocomotion was accompanied by an increased average speed in the MK801 group, t-test, ∗∗∗p = 0.0001. (D) Representative tracking plots from Veh and Ket rats after 5 min of exposure to the elevated plus maze. (E) MK801 treatment induces a reduction in the percentage of open arm entries compared to the Veh group, t-test, ∗p < 0.01. (F) The total distance traveled tested in this paradigm remained unchanged, t-test, p > 0.05. (G) Quantitative analyses show that slices from MK801 treatment have a significant decrease frequency of sIPSC compared to those from Veh rats and increased amplitude of sIPSC (H) (t-test, ∗p < 0.05). (I) Paired-pulse responses superimposed after subtraction of the first pulse at 30, 70, 100, and 300 ms ISIs. Slices from MK801treatment rats showed an increase of paired pulse ratio at intervals equal to 30 ms compared to the Veh group (Repeated measures ANOVA/Bonferroni post hoc test, ∗p < 0.05. (J) Frequency of mIPSC from MK801 treatment was also reduced compared to the Veh group. Moreover, mIPSC amplitude showed a significant increase (K) (t-test, ∗p < 0.05). (L) Synaptic responses evoked by a burst of 20 stimuli at 10 Hz. Depression induced by repetitive stimulation did not changes in its magnitude for slices from MK801 treatment compared to the Veh-treated group (Repeated measures ANOVA/Bonferroni post hoc test, p > 0.05). Data are the mean ± SEM. Number of animals is indicated in parentheses or within bars. [file Image_3.TIF]

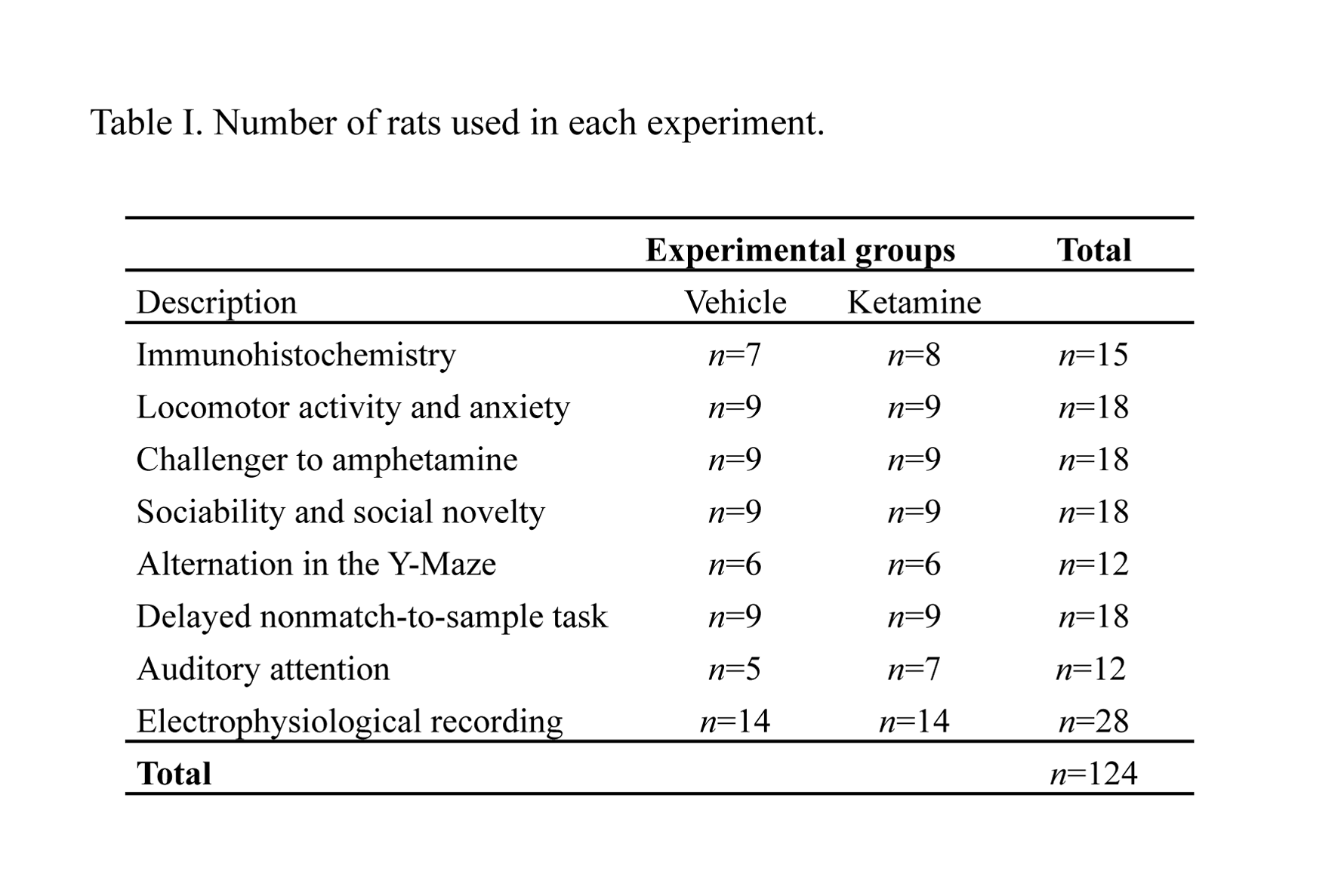

Supplement: TABLE S1 — Number of animals used in each experiment: the table shows the total animals used in each experiment. [file Image_4.TIF]
